# Phylogenetic and functional diversity of metagenomic libraries of phenol degrading sludge from petroleum refinery wastewater treatment system

**DOI:** 10.1186/2191-0855-2-18

**Published:** 2012-03-27

**Authors:** Cynthia C Silva, Helen Hayden, Tim Sawbridge, Pauline Mele, Ricardo H Kruger, Marili VN Rodrigues, Gustavo GL Costa, Ramon O Vidal, Maíra P Sousa, Ana Paula R Torres, Vânia MJ Santiago, Valéria M Oliveira

**Affiliations:** 1 Microbial Resources Division, Research Center for Chemistry, Biology and Agriculture (CPQBA), Campinas University - UNICAMP, CP 6171, CEP 13081-970, Campinas, SP, Brazil; 2 Department of Primary Industries, BioSciences Research Division, 1 Park Dr, Bundoora, Melbourne, Vic 3083, Australia; 3 Department of Enzymology, University of Brasilia (UnB), Brasilia, Brazil; 4 Division of Organic and Pharmaceutical Chemistry, Research Center for Chemistry, Biology and Agriculture (CPQBA), Campinas University, Campinas, Brazil; 5 Laboratory of Genomics and Expression, Department of Genetics and Evolution, Institute of Biology, Campinas University - UNICAMP, CEP 13083-970, Campinas, SP, Brazil; 6 PETROBRAS R&D Center, University City, Square 7, Ilha do Fundão, Rio de Janeiro, RJ, Brazil

**Keywords:** Membrane bioreactor, Fosmid library, Pyrosequencing, Microbial diversity, Metabolic profile

## Abstract

In petrochemical refinery wastewater treatment plants (WWTP), different concentrations of pollutant compounds are received daily in the influent stream, including significant amounts of phenolic compounds, creating propitious conditions for the development of particular microorganisms that can rapidly adapt to such environment. In the present work, the microbial sludge from a refinery WWTP was enriched for phenol, cloned into fosmid vectors and pyrosequenced. The fosmid libraries yielded 13,200 clones and a comprehensive bioinformatic analysis of the sequence data set revealed a complex and diverse bacterial community in the phenol degrading sludge. The phylogenetic analyses using MEGAN in combination with RDP classifier showed a massive predominance of Proteobacteria, represented mostly by the genera *Diaphorobacter, Pseudomonas, Thauera *and *Comamonas*. The functional classification of phenol degrading sludge sequence data set generated by MG-RAST showed the wide metabolic diversity of the microbial sludge, with a high percentage of genes involved in the aerobic and anaerobic degradation of phenol and derivatives. In addition, genes related to the metabolism of many other organic and xenobiotic compounds, such as toluene, biphenyl, naphthalene and benzoate, were found. Results gathered herein demonstrated that the phenol degrading sludge has complex phylogenetic and functional diversities, showing the potential of such community to degrade several pollutant compounds. This microbiota is likely to represent a rich resource of versatile and unknown enzymes which may be exploited for biotechnological processes such as bioremediation.

## Introduction

Wastewater treatment plants (WWTP) represent habitats of continuous change in chemical composition (Szczepanowski *et al. *2008). In oil refineries, the wastewater influent differs daily in terms of concentrations and composition of pollutant compounds, including light fraction aliphatic and aromatic petroleum hydrocarbons, organochlorines originated from cooling liquids used in the industrial process (Stepnowski *et al. *2002) and other compounds such as phenol, chlorides, sulphides, sodium hydroxide, ammonia and heavy metals (Braile 1979; Mariano 2001). Phenols and derivatives are prominent pollutants in these wastes. These compounds are widely used as raw materials in the petrochemical industry and in oil refineries, for example in the washing and conditioning of alkaline or acid products. The increasing presence of phenols in the environment represents a serious ecological problem due to toxicity hazard for living creatures, including micro-organisms (Ojumu *et al. *2005; Barrios-Martinez et al. 2006). Besides, the presence of phenols reduces significantly the biological degradation of the other compounds.

Several processes are used to eliminate phenolic compounds from industrial wastewater, but the biological treatments have been preferred for large-scale removal. However, this is not an easy task because of the proper toxicity of phenol towards microorganisms (Barrios-Martinez et al. 2006). In this sense, the monitoring of the microbiota is very important for efficient performance of biological treatment systems.

Traditionally, the diversity of microbial communities has been accessed by means of cultivation-based techniques or optical microscopy (Henze *et al. *1997; Ojumu *et al. *2005; Chang *et al. *2005). Although very useful for taxonomic, physiological and genetic studies, culture-based techniques are insufficient for a more precise characterization of the functional and phylogenetic diversity of microbial communities, since it is now well known that only a small fraction (0.1 to 10%) of the microbial diversity in nature can be recovered in the laboratory (Torsvik *et al. *1990; Amann et al. 1995).

In the last decade molecular cultivation-independent techniques have allowed the access to yet uncultivated microorganisms in several environmental niches (Handelsman 2004; Sleator *et al. *2008), providing significant insights into bacterial communities in wastewater treatment processes (Sercu *et al. *2006; Miura *et al. *2007; Zang *et al. *2008; Silva *et al. *2010a; Silva *et al*. 2010b). Additionally, with the development of metagenomic approaches, the discovery and exploration of new microbial groups and functions have been accelerated (Handelsman 2004; Steele *et al. *2009).

Nonetheless, molecular studies of microbial communities using Sanger sequencing have been limited by the number of sequences that can be obtained (Zhang *et al. *2009). Microbial ecology studies based on 16S rRNA gene libraries have generally shown an underestimated bacterial diversity (Zhou *et al. *2001; Zhang *et al. *2009; Silva *et al. *2010b). The recent application of next-generation sequencing technologies, such as pyrosequencing, has allowed one to obtain a huge number of sequences, usually sufficient to reveal the complexity of a microbial community in a given sample.

There are a few studies reporting the use of pyrosequencing for the phylogenetic and functional analysis of microbial communities in sludge from wastewater treatment processes. Some authors used this tool to look for plasmids with antibiotic resistance in sewage wastewater (Szczepanowski *et al. *2008; Schlüter *et al. *2008). Other studies employed 454-FLX pyrosequencing to investigate the microbial community from activated sludge of a domestic sewage wastewater treatment plant (Sanapareddy *et al. *2009) or for a comprehensive understanding of the pathogen richn ess and abundance in metagenomic DNA samples derived from sewage sludge, composting and agricultural soil (Billy *et al. *2010).

In this work we used the pyrosequencing approach followed by bioinformatics analysis to carry out an investigation of metagenomic libraries of phenol degrading sludge derived from petroleum refinery WWTP, allowing us to have a snapshot of the phylogenetic and functional microbial diversity selected after phenol enrichment of sludge.

## Materials and methods

### Sampling

Sludge samples were collected from two different refinery wastewater treatment plants of the petroleum producer Petrobras (Brazil). The first sludge sample, named MBR1 and originated from a laboratory-scale membrane bioreactor (2 L) (MBR), was performed with continued aeration and collected after a 30-day period of high phenolic load feeding (68.5 mg L^-1^), as previously described by Viero and collaborators (Viero et al. 2008).

The second sample, named MBR2, was collected from a pilot submerged aerobic membrane bioreactor, previously described by Silva *et al. *(2010b), that has been in continuous operation for 18 months and is part of the industrial WWTP of Petrobras Refinery (Brazil). However, a phenol acclimation step was done to increase the possibility of finding genes related with phenol degradation. The sludge sample MBR2 was acclimatized in batch-culture for a 30 day-period to 1.0 g L^-1 ^of phenol (Merck, USA) and then used for the metagenomic library construction. The acclimation step was performed, in triplicate, using 2.0 g L^-1 ^of sludge, which was centrifuged and placed into an Erlenmeyer flask containing 300 mL of an initial rich nutrient medium (2.75 g L^-1 ^K_2_HPO_4_, 2.25 g L^-1 ^KH_2_PO_4_, 0.1 g L^-1 ^NaCl, 1.0 g L^-1 ^(NH_4_)_2_SO_4_, 0.2 g L^-1 ^MgCl_2_.6H_2_O, 0.01 g L^-1 ^CaCl_2 _and 1 g L^-1 ^yeast extract as carbon source). These flasks were incubated at ambient temperature at 150 rpm. The initial carbon source was gradually diminished and replaced with phenol, in the proportion of 0.5 g L^-1 ^less of yeast extract for each 0.2 g L^-1 ^increment of phenol, until the yeast extract was totally eliminated. The modification of the initial medium composition was performed every three days, where an aliquot of the previous culture medium (10%) was added as inoculum to a new culture medium containing more phenol. The acclimatizing process was monitored through phenol quantification using gas chromatography. The sludge was collected after the microorganisms were considered totally adapted to 1.0 g L^-1 ^phenol, when 100% phenol was removed in less than 24 hours.

### Nucleic acid extraction and metagenomic fosmid library construction

High molecular weight DNA extraction from sludge samples was carried out using the protocol previously described by Silva *et al. *(2010a). Fosmid libraries were constructed using the CopyControl™ HTP Fosmid Library Production Kit (Epicentre, USA), according to the manufacturer's instructions. The DNA fragment size of interest was selected prior to the construction of the metagenomic libraries. The extracted DNA was run on a 1% low-melting agarose gel (Sigma, USA) and submitted to pulsed field gel electrophoresis (*Pulsed-field CHEF DRIII System - *BioRad- USA) at 0.5 s switch time, 9 Vcm^-1^, 120° included angle, 5 h at 14°C. DNA fragments of about 25-50 kb were isolated from the agarose gel, submitted to the end-repair reaction and ligated into the pCC2Fos fosmid vectors. These vectors were packed and transfected into *Escherichia coli *EPI300TM-T1^R ^cells, which were plated onto Luria-Bertani (LB) agar medium containing chloramphenicol (12.5 μg/mL) and incubated overnight at 37°C. Two metagenomic libraries were constructed, one for each sludge sample (MBR1 and MBR2).

For the validation of the metagenomic libraries, six fosmid clones were randomly selected from each library. The fosmid DNA of each clone was extracted using the FosmidMax DNA Purification kit (Epicentre, USA), according to the manufacturer's protocol, and then digested using 10 U *NotI *(Promega, USA) at 37°C overnight. The band profiles of fosmid clones were checked in preparative pulsed field gel electrophoresis (*Pulsed-field CHEF DRIII System - *BioRad- USA) at angle 120°, 6 Vcm^-1^, 1 s - 12 s switch time, 10.5 h at 14°C.

### Extraction of fosmid DNA pools and pyrosequencing

Clones derived from both metagenomic libraries were grown in 96-well plates containing 1 mL of LB medium and chloramphenicol (12.5 μg/mL) for 17 h at 37°C at 180 rpm. For fosmid extractions, an aliquot of 500 μL was aliquoted from each well of ten 96 well-plates and pooled, totaling a final volume of approximately 500 mL for each extraction. Each pool, containing 960 clones, was extracted using the Large-Construction Kit (Qiagen, USA), according to the manufacturer's protocol.

Fosmid DNA extracted from all pools for both libraries was then combined to produce a single DNA sample, a master pool of 5 μg DNA, for subsequent pyrosequencing. This master pool was firstly nebulized, for 2 minutes, to produce DNA fragments with average size of 500 bp. The DNA fragment ends were polished and purified to remove fragments < 500 bp and the Roche 454 GS FLX Titanium sequencing (454 Life Sciences Branford, CT, USA) was performed according to 454/Roche GS-FLX protocols.

### Assembly and sequence analysis

The sorting and trimming of the metagenomic data, based on quality and size of the reads, as well as the contig assembling were done using the 454 Newbler assembler (version 2.0.01.14) for *Genome Sequencer FLX *(Roche, USA).

For the phylogenetic classification, the metagenomic data were compared using BLASTn (E-value ≤ 1e-2) against all 16S rRNA sequences deposited in the European Ribosomal RNA Database (http://bioinformatics.psb.ugent.be/webtools/rRNA/). Then, all reads present in the metagenomic data that matched with any rRNA sequence were selected and submitted to a phylogenetic classification using the RDP naïve Bayesian rRNA Classifier tool, at 80% confidence threshold, and E-value of ≤ 1e-2, available on the Ribosomal Database Project's Pyrosequencing Pipeline website.

The contig and singlet sequences were analyzed with MEGAN software and the MG-RAST platform. These sequence sets were submitted to protein BLAST for sequence matching using the NR database (http://blast.ncbi.nlm.nih.gov/Blast.cgi). The resulting BLASTp file was then analyzed using MEGAN metagenomic software (Huson *et al. *2007). MEGAN software uses a homology-matching algorithm to generate a phylogenetic tree based on the NCBI taxonomic database.

**Figure 1:**
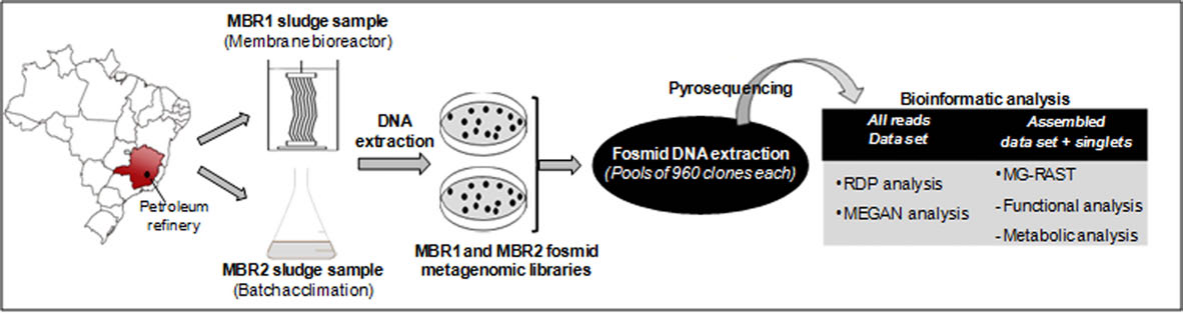
**Overview of the experimental approach employed in this work**.

The metabolic pathway classification of contig and singlet sequences was done using the MG-RAST platform (http://metagenomics.nmpdr.org/metagenomics.cgi?page=Logout). The data were submitted as text file (.txt) for annotation using the subsystems technology. In this approach, reads are classified in a hierarchical structure in which all genes required for a specific function are grouped into subsystems (Aziz *et al. *2008). An overview of the experimental approach used in this study is illustrated in Figure 1.

### Nucleotide sequence accession number

The raw data set from the fosmid libraries is available from the NCBI Short Read Archive (SRX038779.2).

### GC-MS analysis of phenol of acclimated sample

To verify if the MBR2 sample microorganisms were degrading phenol after acclimation process, the phenol concentration was determined by 1:1 extraction with CH_2_Cl_2 _according to the procedures described in Standard Methods (1998). Phenol quantitation was done using a calibration curve constructed from a fortified matrix in three levels. Growth culture samples were filtered through a 0.45 μm membrane filter to remove suspended biomass before analysis. One microliter of extracted sample was subsequently injected into a gas chromatography/mass spectrometry (model 6890 N, Agilent, USA) equipped with a 5975 selective mass detector and 7683B automatic injector. The sample components were separated in the HP-5 MS (30 m × 0.25 mm × 0.25 μm) capillary column using the following temperature programming: 60°C (3°/min), 240°C/10 min. The injector temperature was 220°C and the interface temperature was 250°C. Split ratio was 1/100 and the monitoring fragments were 94 (quantification ion), 66 and 65 (identification ions).

## Results

### MBR2 sludge acclimation

The acclimation step was done only for the MBR2 sample, since this sludge sample originated from a membrane bioreactor operating without high phenol concentration. The biomass was progressively acclimated to a maximum phenol concentration of 1.0 g L^-1 ^for 30 days, and after this period the biomass was able to degrade 1.0 g L^-1 ^of phenol in less than 24 hours.

The biomass acclimation was checked by monitoring phenol degradation using visual and chromatographic analysis during 30 days. The visual analysis consisted of observation of microbial aggregates (flocculation) and the production of yellow pigments in the culture medium, which, according to Harayama *et al. *(1992), is indicative of phenol biodegradation through the *meta*-cleavage pathway. The yellow color comes from the product of cathecol cleavage reaction, 2-hydroxymuconic semialdehyde. Additionally, the phenol degradation was monitored by chromatographic experiments, which demonstrated that 90% of phenol was degraded in the first 12 hours, and in less than 24 hours 1.0 g L^-1 ^of phenol was totally removed by the acclimated biomass.

### Metagenomic fosmid libraries and pyrosequencing

Two metagenomic fosmid libraries were made using high molecular weight DNA extracted from the total microbial community of MBR1 and MBR2 sludge samples, yielding 10,000 and 3,200 clones, respectively. The libraries were validated by enzyme digestion in order to check the diversity of clones obtained. Pulsed field gel electrophoresis revealed distinct band profiles for the six randomly selected clones from each library, with the vector band present in the same position for all clones, confirming the occurrence of different insertion events.

The 13,200 clones obtained from both libraries were then submitted to fosmid DNA extraction in pools and subsequent pyrosequencing. A single pyrosequencing run was done for two libraries, yielding 322,742 reads with an average sequence length of 263 bp, the largest sequence being 735 bp. The metagenomic data was assembled using Newbler (version 2.0.01.14), which employs algorithm attempts to combine individual sequence reads into longer contigs. The assembly recruited 108,000 sequences, which resulted in 22,267 contigs and 99,786 singletons.

### Phylogenetic composition of phenol degrading sludge by MEGAN

**Figure 2:**
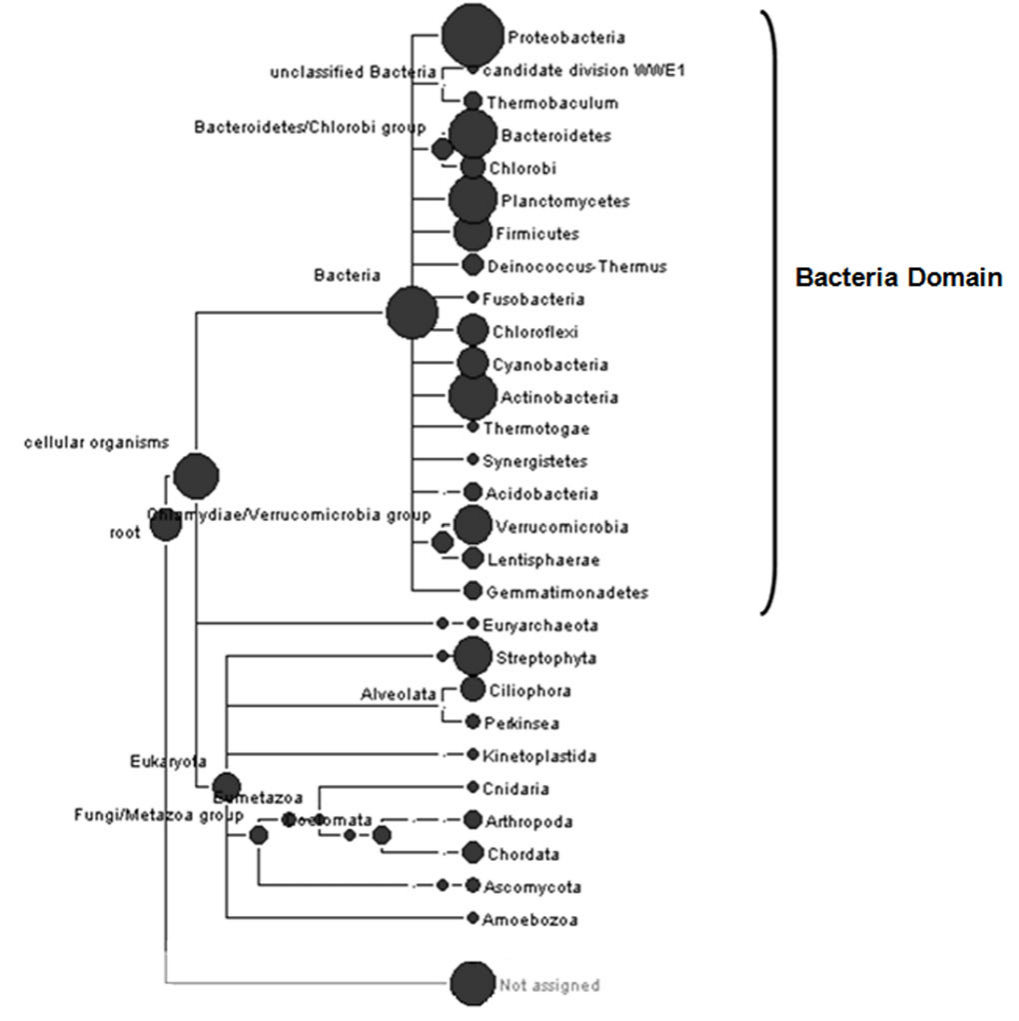
**Phylogenetic tree of all reads derived from the enriched refinery sludge community by MEGAN analysis**.

Community taxonomic analysis of metagenomic data was performed using MEGAN (Metagenome analysis software). This software generated a phylogenetic tree based on the NCBI taxonomic database, in which the size of each circular node is proportional to the number of assignments at the particular taxonomic level. The phylogenetic tree showed that the majority of the metagenomic sequences were affiliated to the Bacteria domain (64%) and the remaining sequences were distributed between the Archaea and Eukarya domains (Figure 2). The eukaryotes were represented mainly by fungi and protozoa, common organisms present in the sludge of wastewater treatment system (Jiang *et al. *2005; Oliveira *et al. *2009). A considerable percentage of metagenomic sequences (34%) were unaffiliated. Possible these sequences are distantly related with any known sequence deposited in the public databases.

The Bacteria domain was represented by sixteen different phyla encompassing Proteobacteria, Candidate Division WWE1, Bacteroidetes/Chlorobi, Planctomycetes, Firmicutes, Deinococcus-Thermus, Fusobacteria, Chloroflexi, Cyanobacteria, Actinobacteria, Thermotogae, Synergistetes, Acidobacteria, Verrucomicrobia, Lentisphaerae and Gemmatimonadetes (Figure 2). Among these, the most abundant were Proteobacteria (66%), followed by Bacteroidetes (7%), Actinobacteria (5.5%), Planctomycetes (4.9%) and Verrucomicrobia (1.3%). This finding supports previous reports of our research group that proteobacteria is a predominant phylum in sludge samples (Silva *et al. *2010a; Silva *et al. *2010b), suggesting that these organisms play key roles in biodegradation processes that take place in petroleum refinery wastewater treatment systems.

**Figure 3:**
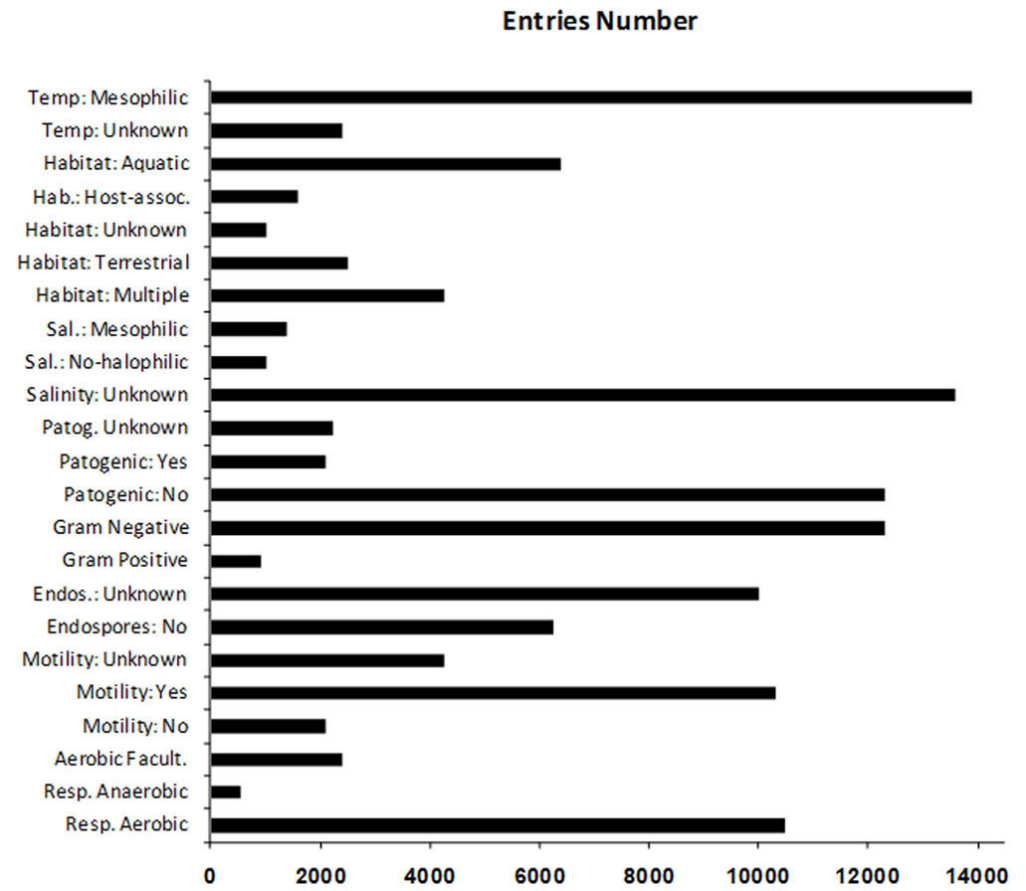
**Attributes of the metagenomic library of phenol degrading sludge from membrane bioreactor obtained by MEGAN analysis of all reads**.

The metagenomic data analysis using MEGAN also yielded a profile of attributes for the bacterial community, in which the majority of the bacterial species were shown to be gram-negative, aerobic, motile, non-pathogenic, aquatic and mesophilic (Figure 3). These characteristics are in accordance with those displayed by the vast majority of proteobacteria detected in the sludge of WWTPs (Miura *et al. *2007; Kraigher *et al. *2008; Sanapareddy *et al. *2009, Silva et al. 2010a, Silva et al. 2010b).

### Phylogenetic classification of metagenomic sequences

**Figure 4:**
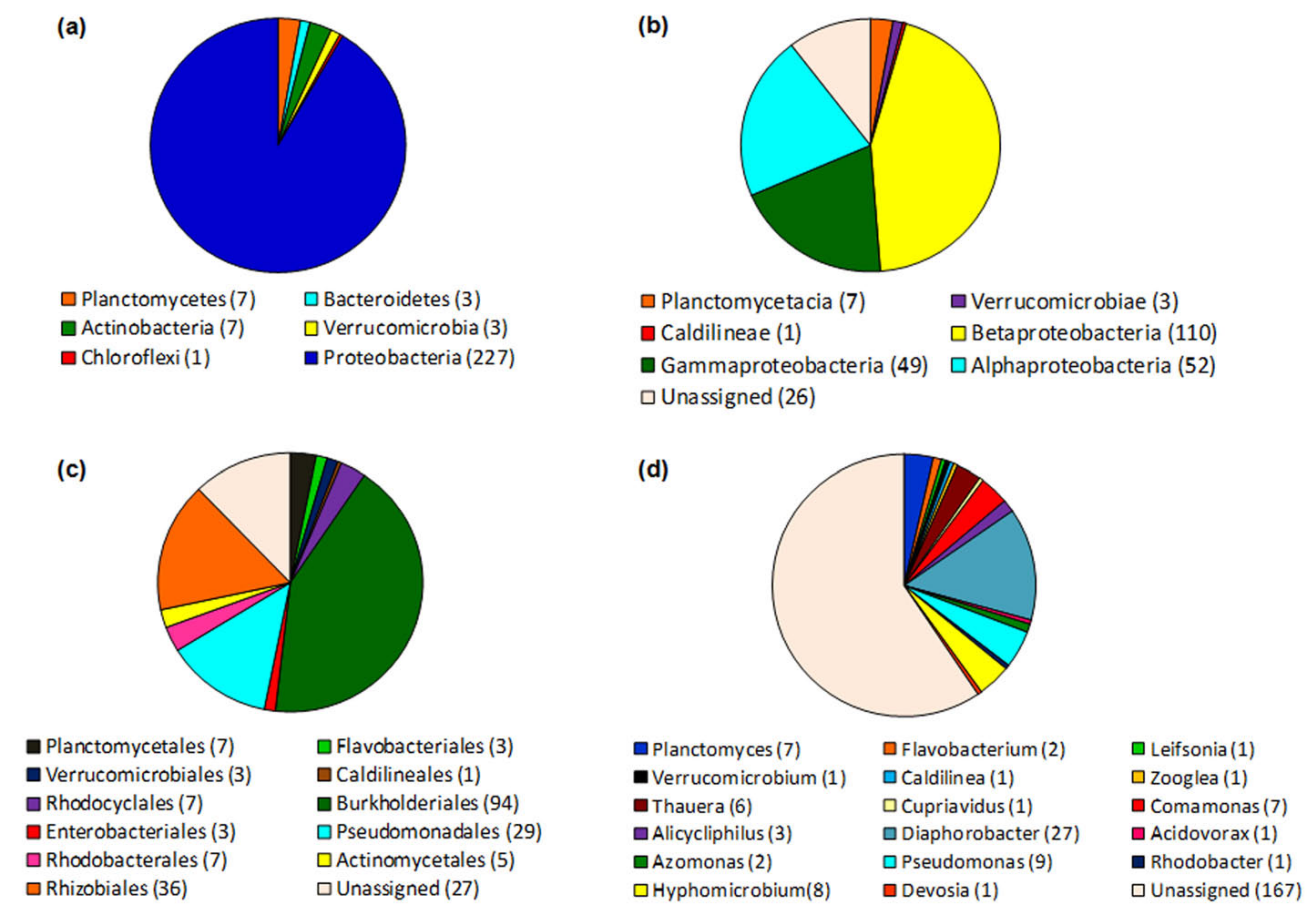
**Classification of the 16S rRNA gene sequences of metagenomic library of phenol degrading sludge from membrane bioreactor using the RDP database**. (**a**) Phylum, (**b**) Class, (**c**) Order and (**d**) Genus. Sequences that were not classified at the Phylum level were excluded.

The comparison of all metagenomic sequences with the European Database allowed the selection of 6,099 sequences presenting some 16S rRNA gene similarity, this dataset was submitted to automatic annotation using the RDP Classifier. This algorithm platform uses Bayesian statistics to assign 16S rRNA genes sequences to known taxa. A set of 247 reads was annotated at least to the phylum level based on known 16S rRNA genes sequences from RDP database at an E-value cutoff ≤ 0.01. The selected size of sort reads in the pyrosequencing data was 100 nt at least. This sequence length was chosen based on the work of Liu *et al. *(2007) showing that reads as short as 100 bases are long enough to accurately characterize taxa. The annotation results revealed the presence of six different phyla in the sludge samples under study, named Proteobacteria, Actinobacteria, Chloroflexi, Planctomycetes, Verrucomicrobia and Bacteroidetes (Figure 4). Despite the fact that this analysis uses only 16S rRNA gene sequences for the classification, most of the results were consistent with the MEGAN analysis (Figure 2).

At the phylum level, the observed taxa were dominated by representatives of Proteobacteria, which accounted for 91% of the classified sequences (Figure 4a). From these, about 45% were affiliated to class Betaproteobacteria (Figure 4b). The majority of the 16S rRNA sequences were classified at the order level and, distributed across 11 different orders with Burkholderiales (Betaproteobacteria) the most abundant, followed by Rhizobiales (Alphaproteobacteria) and Pseudomonadales (Gammaproteobacteria) (Figure 4c). At the genus level, the sequences could be classified in 17 known genera of which *Diaphorobacter*, *Pseudomonas, Hyphomicrobium, Comamonas, Planctomyces *and *Thauera *were the most prevalent. As classification of the sequences moved from phylum to genus, fewer sequences could be classified with a RDP confidence score of at least 80%, and 67% of the sequences were considered unassigned (Figure 4d), confirming previous reports on the huge bacterial diversity of wastewater treatment sludges (Miura *et al. *2007; Sanapareddy *et al. *2009; Silva *et al. *2010a; Silva *et al. *2010b).

### Functional analysis of the metagenome phenol degrading sludge

**Figure 5:**
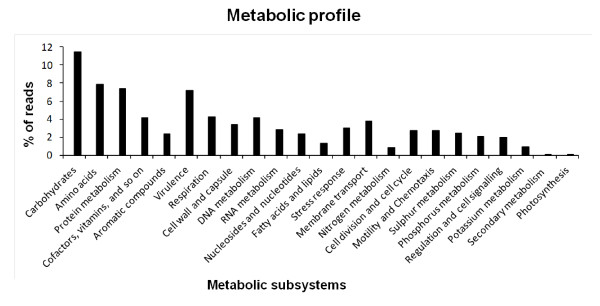
**Metabolic profile of phenol degrading sludge metagenomic libraries datasets using MG-RAST platform**.

The sequences were functionally annotated based upon MG-RAST plataform using the SEED database. The functional analysis allowed the classification of 42.8% of the metagenomic sequences into several subsystems with the majority of them related to the metabolism of carbohydrates, aromatic compounds, proteins, DNA, amino acids and derivatives, virulence, respiration and cofactors. By contrast, some subsystems were under-represented in the metagenomic data, including sequences involved in photosynthesis, secondary metabolism, macromolecular synthesis and nitrogen metabolism (Figure 5). Despite of the low number of reads assigned to the nitrogen metabolism, the majority of these sequences represented genes coding enzymes linked to processes such as nitrate and nitrite ammonification and denitrification, which are considered important processes for the removal of complex nitrogen compounds in wastewater treatment. An interesting result was observed for the virulence subsystem (Figure 5), which accounted for a high number of reads. Annotation of such reads revealed that almost 50% of them were related to antibiotic and toxic compounds resistance genes, which are extremely important features for microbial survival and adaptation in polluted environments.

### Analysis of metagenome for phenol degradation

The phenol can be aerobically degraded for two different pathways, *ortho*- or *meta*-pathway. The aromatic ring is initially monohydroxilated in the adjacent carbon of a hydroxyl group by the enzyme phenol hydroxylase (EC 1.14.13.7) resulting in catechol, which is in turn cleaved by either *ortho*- or *meta*-cleavage pathway. In case of the *ortho*-pathway, the ring is cleaved by the catechol 1,2-dioxygenase enzyme (EC 1.13.11.1), leading to the initial formation of succinyl-CoA and acetyl-CoA. In the *meta*- pathway, the catechol is cleaved by the catechol 2,3-dioxygenase enzyme (C23O), leading to the formation of pyruvate and acetaldehyde (Marimaa *et al*. 2006).

**Figure 6:**
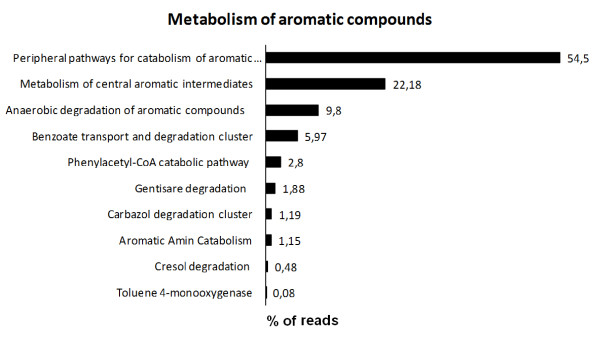
**Percentage of sequences associated to aromatic compound metabolism of phenol degrading sludge metagenomic libraries from membrane bioreactor by MG-RAST platform**. Total of aromatic compounds metabolism reads = 3800.

In MG-RAST analysis, the aromatic compound utilization profile of phenol degrading sludge was dominated by proteins annotated as subsystems of peripheral pathway for catabolism of aromatic compounds and metabolism of central aromatic intermediates, besides benzoate degradation, cresol degradation and toluene 4-mooxygenase, that are also important pathways involved in the catabolism of pollutant compounds found in refinery wastewater (Figure 6).

The subsystem of peripheral pathway for catabolism of aromatic compounds encompassed the proteins involved with the degradation of phenol and derivatives, such as phenol hydroxylase (EC 1.14.13.7) (78 reads) and enzymes involved in the biphenyl degradation (75 reads). The subsystem of metabolism of central aromatic intermediates, although mainly represented by the homogentisate pathway, involved in the catabolism of aromatic rings, contained the proteins involved in the *ortho- *and *meta*-pathway of phenol degradation, such as catechol 1,2-dioxygenase (EC 1.13.11.1) (9 reads) and catechol 2,3- dioxygenase (EC 1.13.11.2) (23 reads), respectively.

These analyses showed that the genes related to phenol degradation have been well sampled in the phenol degrading sludge, revealing that the phenol enrichment was successful. Further studies will be carried out to verify if these genes represent new sequences coming from uncultivated microorganisms.

### Analysis of the metabolic potential for organic matter removal

This analysis was done to investigate the presence of some important functions related to the biological treatment performance in the microbial sludge under study after the phenol enrichment.

The potential metabolic functions for organic matter removal were established by comparison of all pyrosequencing-derived sequences from phenol degrading sludge to the KEGG maps using functional assignments from SEED analysis (Meyer *et al. *2008). Within the KEGG categories, matches were separated into different subcategories and several sequences were assigned to more than one subcategory. The most represented categories were metabolism of energy, amino acids, carbohydrates, cofactors and nucleotides. These categories showed a high number of distinct Enzyme Commissions (EC) belonging to a specific metabolic pathway. The EC number is the common name for IUBMB's Enzyme Nomenclature System, and plays a key role in classifying enzymatic reactions and linking the enzyme or protein genes to reactions in metabolic pathways (Yamanishi *et al. *2009).

Among the KEGG categories found, the energy metabolism and the biodegradation of xenobiotics are emphasized, since they constitute important processes for wastewater biological treatment, such as removal of organic matter, including pollutant compounds, from the petroleum refining process. About 4,000 metagenomic sequences were related to the energy metabolism category, which involved homology to known genes of carbon fixation, oxidative phosphorylation, nitrogen metabolism and CO_2 _fixation (Table 1). On the other hand, the number of genes that matched with methane metabolism subcategories was low, which is consistent with the aerobic biological treatment. In the sulfur metabolism subcategory, most sequences showed similarities with enzymes related to sulfur assimilation pathways, such as cystathionine synthase, homoserine acetyltransferase, homoserine succinyltransferase and sulfate kinases. In the nitrogen metabolism subcategory, the majority of the sequences were related with nitrate and nitrite reductase, enzymes involved in nitrogen assimilation pathways. A total of 2,495 metagenomic sequences were assigned to the xenobiotic biodegradation category, which showed, in general, a more even distribution among the subcategories when compared to the previous category. Exceptions were the benzoate degradation via CoA ligation subcategory, which presented the highest number of matches, and the carbazole degradation subcategory, which in turn showed the lowest number of matches (Table 1).

**Table 1:** Number of sequences showing homology to genes associated with KEGG pathways in the categories "carbohydrate metabolism", "biodegradation of xenobiotics" and "energy metabolism"

KEGG category	Distinct ECs	**ECs found in the data **(% abundance)	N° of matches
***Energy metabolism***	***173***	***92 (53.5%)***	***3,857***

Carbon fixation	25	21 (84%)	986

Methane metabolism	33	12 (36.4%)	202

Nitrogen metabolism	58	30 (51.7%)	739

Oxidative phosphorylation	17	09 (52.9%)	972

Photosynthesis	03	01 (33%)	168

Redutive carboxylate cycle (CO2 fixation)	13	13 (100%)	531

Sulfur metabolism	30	12 (40%)	267

***Biodegradation of Xenobiotics***	***217***	***66 (30.4%)***	***2,495***

DDT degradation	10	03 (30%)	74

1,2-Dichloroethane degradation	05	04 (80%)	85

1,4-Dichlorobenzene degradation	22	06 (27.3%)	74

2,4-Dichlorobenzoate degradation	29	03 (10.3%)	18

3-Chloroacrylic acid degradation	04	04 (100%)	185

Atrazine degradation	12	04 (33.3%)	24

Benzoate degradation via CoA ligation	45	23 (51%)	994

Benzoate degradation via hydroxylation	50	18 (36%)	158

Biphenyl degradation	13	03 (23.1%)	71

Caprolactam degradation	22	10 (45.5%)	131

Carbazole degradation	11	01 (9.1%)	03

Ethylbenzene degradation	09	03 (33.3%)	207

Fluorene degradation	13	03(23.1%)	64

Naphthalene and anthracene degradation	21	03 (14.3%)	188

Styrene degradation	21	09 (42.9%)	73

Tetrachloroethene degradation	07	01 (14.3%)	57

Toluene and xylene degradation	22	07 (31.8%)	32

γ-Hecachlorocyclohexane degradation	24	07 (29.2%**)**	57

***Carbohydrate metabolism***	***576***	***284 (49.3%)***	***9,901***

Aminosugars metabolism	41	25 (61%)	589

Ascorbate and aldarate metabolism	43	18 (41.9%)	367

Butanoate metabolism	53	34 (64.2%)	1,802

C5-Branched dibasic acid metabolism Fructose	17	03 (17.6%)	126

and mannose metabolism	66	28 (42.4%)	591

Galactose metabolism	37	19 (51.4%)	529

Glycolysis/Gluconeogenesis	42	29 (69%)	858

Glyoxylate and dicarboxylate metabolism	58	30 (51.7%)	676

Inositol metabolism	09	07 (77.8%)	90

Inositol phosphate metabolism	30	05 (16.4%)	34

Nucleotide sugars metabolism	34	18 (52.9%)	517

Pentose and glucuronate interconversions	56	27(48.2%)	229

Pentose phosphate pathway	39	31 (79.5%)	846

Propanoate metabolism	49	34 (69.4%)	1032

Pyruvate metabolism	65	39 (60%)	1,117

Starch and sucrose metabolism	73	33 (45.2%)	498

In addition, the analysis of the carbohydrate metabolism category showed a high number of matches (9,387 sequences) related to genes involved in several subcategories for utilization of carbohydrates (Table 1). These results demonstrated the broad capability of microorganisms from refinery sludge to metabolize distinct sugar sources.

## Discussion

This is the first report of a pyrosequencing approach for a broad phylogenetic and metabolic diversity analysis of metagenomic fosmid libraries derived from phenol degrading sludge samples of petroleum refinery WWTP. Sequencing of the metagenomic libraries has allowed us to have deeper insight to the complex metagenome of the phenol degrading sludge samples, with thousands of reads assigned to different taxa and metabolic categories driving the functioning of the microbial community in the membrane bioreactors.

Based on previous studies of the use of short sequence reads (~90 bp) to accurately classify microbial communities (Liu *et al. *2007; Sanapareddy *et al. *2009), this work used MEGAN and RDP Classifier tools for obtaining a microbial phylogenetic profile of the metagenomic data. The results obtained using both tools were consistent and showed that the most abundant phyla in the metagenomic data were similar, despite the fact that the MEGAN analysis is based on all metagenomic reads, while the RDP Classifier is based on only the 16S rRNA gene reads.

Results of RDP classifier were compared to a previous report by Silva and collaborators (2010a), where the authors used the phenol degrading sludge sample (MBR1) to construct a 16S rRNA gene library. The comparison of the two libraries showed that Proteobacteria was the predominant phylum, but the richness of phyla was higher in the metagenomic dataset, which contained 6 different phyla, whereas the 16S rRNA library revealed only 3 phyla for the MBR 1 sample. However, the comparison between deeper levels, such as order and genus, showed that the richness between both libraries is similar, although the groups found were different. Probably, these differences can be explained by the bias inherent to each method, e.g. pyrosequencing and 16S rRNA gene library, used to survey the bacterial diversity. Nonetheless, in this case, the data obtained using both approaches can be considered complementary in order to depict a bacterial diversity scenario of the phenol degrading sludge.

The high abundance of the Proteobacteria group in microbial communities from wastewater treatment samples was also observed in several other studies using PCR-based experiments targeting the 16S rRNA gene (Miura *et al. *2007; Ahmed *et al. *2007; Li *et al. *2009; Sanapareddy *et al. *2009; Silva *et al. *2010a; Silva *et al. *2010b). The predominance of Proteobacteria in such environments could be explained by the fact that this phylum comprises the most phylogenetically diverse group in the Bacteria Domain, known to be metabolically versatile, including aerobic and facultative aerobic bacteria (Madigan *et al. *2008). These are quite interesting characteristics for microorganisms inhabiting wastewater treatment plant, an environment showing great daily variations in the composition and concentration of pollutant compounds. These results demonstrate that phylogenetic studies based on pyrosequencing of metagenomic fosmid libraries can give broad and reliable information about the predominant microbial groups present in the microbial community sampled.

Within the Proteobacteria, a few genera containing important species, e.g. *Pseudomonas putida *(Marques and Ramos 1993, Gonzales *et al. *2001), *Thauera aromatica *(Breinig et al. 2000), *Thauera aminoaromatica*, *Thauera phenylacetica *(Mechichi *et al. *2002), *Thauera *sp. DNT-1 (Shinoda *et al. *2004) and *Comamonas testosterone*, are able to utilize different kinds of aromatic compounds, including phenol, polyphenol, toluene and halobenzoate as carbon sources. Functional studies based on SIP-RNA have revealed that members of *Thauera *genus dominated the phenol degradation process in bioreactor sludges (Manefield *et al. *2002; Valle *et al*. 2004). Additionally, studies based on culturing analyses have showed that *Comamonas testosteroni *can be involved with the metabolism of aromatic compounds, such as phenol and 4-clorophenol (Bae *et al. *1996; Arai *et al. *1998). Basu et al. (2006) verified that *Pseudomonas putida *CSV86 is able to degrade preferentially naphthalene over glucose. Agarry and Solomon (2008) studied batch culture using synthetic phenol and observed that *Pseudomonas fluorescens *was able to degrade phenol in the concentration range of 100-500 mg/L.

A considerable fraction of the metagenomic sequence data was not assigned at the genus level, demonstrating the astonishing microbial diversity present in the sludge from wastewater treatment plants. Similar findings were reported by Sanapareddy *et al. *(2009) when analyzing sewage sludge from biologic treatment system, and these data corroborate other previous studies of complex environments, such as soil and oceans, in which the sequence classification becomes worse as one moves into deeper taxonomic levels (Roesch *et al. *2007; Brown *et al. *2009).

The phylogenetic richness observed reflected the wide metabolic diversity present in the metagenomic data from the WWTP sludge. As expected, genes assigned to the metabolism of carbohydrates, amino acids and proteins were more numerous since they are related to the housekeeping functions of all living organisms. The presence of sequences assigned to functions like nitrogen, phosphor, sulfur and aromatic compounds metabolisms are essential for the high performance of wastewater treatment plant, since they are indicative that the microorganisms from the sludge are degrading and/or assimilating such compounds. High concentrations of these organic compounds, such as ammonia, sulfate, phosphate, phenol may be toxic to human health and cause negative environmental impacts (Nair *et al. *2008).

The metabolic profile revealed a broad set of important genes related to the utilization and mineralization of aromatic and/or xenobiotics compounds, including some key enzymes related to aerobic phenol degradation, such as phenol hydroxylase, catechol 1,2 dioxygenase and catechol 2,3 dioxygenase. A high number of sequences related to benzoate degradation pathway was observed as well, what is coherent with the fact that benzoate is a central intermediary compound in the anaerobic and aerobic metabolism of various aromatic compounds, such as toluene, xylene, fluorene, carbazole and biphenyl (Kim and Harwood 1991). In addition, the anaerobic phenol degradation via carboxylation of phenol to 4-hydroxybenzoate ends in the anaerobic benzoate pathway (Lack and Fuchs 1994). These results suggest that the microorganisms of the sludge under study have potential to degrade phenol and derivatives via aerobic and anaerobic pathway. This versatility is actually a great benefit to the performance of the biological treatment.

Finally, the phylogenetic and metabolic diversities observed are an indicative that the phenol enrichment of the sludge did not affect other important functions besides phenol degradation, which are necessary for the efficient performance of biological treatment systems. Additionally, the metagenome dataset generated by pirosequencing may provide useful sequence information for the characterization of whole catabolic pathways, particularly phenol degrading pathway, that support fundamental key processes occurring in the wastewater treatment plants. Future studies will be conducted aiming at the design and use of probes or primers to detect fosmid clones bearing specific target new genes and pathways related to pollutant compound degradation, thus offering efficient tools for the improvement of bioremediation technologies.

## Competing interests

The authors declare that they have no competing interests.
